# Controlled Growth of ZIF-8 Membranes on GO-Coated α-Alumina Supports via ZnO Atomic Layer Deposition for Improved Gas Separation

**DOI:** 10.3390/membranes14100216

**Published:** 2024-10-14

**Authors:** Nahyeon Lee, Yun-Ho Ahn, Jaheon Kim, Kiwon Eum

**Affiliations:** School of Chemical Engineering, Soongsil University, Seoul 06978, Republic of Korea; nahyunn11@soongsil.ac.kr (N.L.); yhahn@ssu.ac.kr (Y.-H.A.); jaheon@ssu.ac.kr (J.K.)

**Keywords:** metal-organic frameworks, membranes, gas separation, graphene oxide, hollow fibers

## Abstract

This study presents a novel approach for fabricating ZIF-8 membranes supported on α-alumina hollow fibers through the introduction of a graphene oxide (GO) gutter layer and the application of zinc oxide (ZnO) Atomic Layer Deposition (ALD). The method successfully addressed key challenges, including excessive precursor penetration and membrane thickness. The introduction of the GO layer and subsequent ZnO ALD treatment significantly reduced membrane thickness to approximately 300 nm and eliminated delamination issues between the GO layer and the alumina support. The optimized membranes demonstrated enhanced propylene permeance, with values approximately three times higher than those of membranes without GO, and achieved higher separation factors, indicating minimal inter-crystalline defects. Notably, the GO layer influenced the microstructure, leading to an increase in permeance with rising temperatures. These findings highlight the potential of this strategy for developing high-performance ZIF-8 membranes for gas separation applications.

## 1. Introduction

The development of advanced materials for membrane-based separations has become a crucial area of research due to the growing demand for energy-efficient and environmentally sustainable separation technologies [1]. Among various membrane materials, Metal-Organic Frameworks (MOFs) have shown great potential, owing to their unique features, such as tunable pore structures, high surface areas, and the ability to modify chemical functionalities. In particular, Zeolitic Imidazolate Framework-8 (ZIF-8), a subclass of MOFs, has garnered significant attention for gas separation applications, especially in the difficult separation of propane (C_3_H_8_) and propylene (C_3_H_6_), which are vital in the petrochemical sector [2,3].

ZIF-8 consists of zinc ions (Zn^2^⁺) coordinated with 2-methylimidazolate (2 mIm) ligands, resulting in a sodalite (SOD) topology [4,5]. This three-dimensional structure features micropores around 4.0 Å, which are ideally suited for size-selective separations [6]. ZIF-8’s moderate thermal and chemical stability further enhances its suitability for use in certain industrial operating conditions. [7]. Despite these advantages, producing defect-free ZIF-8 membranes with precise control over thickness and uniformity remains a major challenge. Defects, such as inter-crystalline gaps and inconsistent coverage, can significantly impair membrane performance, particularly in gas separation processes that rely on high selectivity and permeance [8].

Conventional methods for ZIF-8 membrane fabrication include solvothermal synthesis, dip-coating, and secondary growth [9]. While these techniques have proven successful in forming ZIF-8 membranes, they often face challenges like long processing times, high precursor usage, and difficulties in controlling membrane thickness and uniformity. Additionally, direct immersion of substrates in precursor solutions can lead to excessive penetration of the precursors into the substrate pores, resulting in thick, uneven membranes that may suffer from poor separation performance due to defects [10].

To overcome these limitations, Rapid Thermal Deposition (RTD) has been proposed as a novel method for synthesizing ZIF-8 membranes [11,12]. RTD utilizes the rapid evaporation of a precursor solution absorbed into the substrate’s pores to form a selective layer. This approach offers several advantages, including reduced precursor consumption, shorter processing times, and improved control over membrane thickness. In our previous work, we successfully fabricated ZIF-8 membranes using RTD, where the precursor solution was absorbed into an α-alumina substrate’s pores, followed by rapid thermal treatment to induce crystallization [10,13]. The resulting membranes displayed uniform thickness and a lack of large-scale defects.

Despite significant progress in the development of ZIF-8 membranes mentioned above, challenges remain in achieving defect-free membranes with controlled thickness and improved separation performance. The knowledge gap lies in the excessive penetration of precursors and delamination issues when using RTD methods. In this work, we hypothesize that introducing a GO gutter layer combined with ZnO ALD will provide better control over precursor distribution and improve membrane stability. Our strategy involves the use of rapid thermal deposition (RTD) to fabricate ZIF-8 membranes on GO-coated α-alumina hollow fibers. The results demonstrate a significant improvement in membrane uniformity, reduced thickness, and enhanced gas separation performance, addressing the limitations of conventional approaches.

## 2. Experimental Section

### 2.1. Materials

α-Alumina powder (1 μm) was provided by Baikowski, Poisy, France. Polyethersulfone, polyvinylpyrrolidone, and N-methyl-2-pyrrolidone (NMP) were sourced from Sigma-Aldrich, St. Louis, MO, USA The epoxy adhesive was acquired from 3M, Saint Paul, MN, USA. 2-Methylimidazole (2 mIm) and zinc acetate dihydrate (Zn(OAc)₂) were also purchased from Sigma-Aldrich. Oxidized graphite (FP 99.95% pure) was obtained from Graphit Kropfmühl AG, Hauzenberg, Germany. Additionally, methanol (MeOH), dimethylacetamide (DMAc), and acetic acid were supplied by Daejung Chemicals, Siheung-si, Republic of Korea. Lastly, deionized (DI) water was produced by Direct-Pure Up(Rephile Bioscience, Republic of Korea).

### 2.2. Methods

#### 2.2.1. α-Alumina Hollow Fiber Preparation

α-Alumina hollow fibers were produced using a dry-jet-wet spinning method, followed by sintering, in accordance with established protocols [14,15]. The dope solution was prepared by intensive stirring for 24 h to ensure homogeneous dispersion. Detailed dope information can be found in Table S1. This solution was then loaded into the spinning apparatus, and a mild vacuum (10 psia) was applied overnight to eliminate most of the air bubbles generated during the agitation of the viscous polymer dope. Fiber spinning conditions are tabulated in Table S1. Post-spinning, the fibers underwent a series of solvent exchanges, first with methanol and then with hexane, before being air-dried at 333 K. The fibers were subsequently subjected to a sintering process at 873 K for 3 h, followed by a second sintering stage at either 1723 K or 1773 K for an additional 6 h. Throughout the heating process, a temperature ramp rate of 2 K/min was consistently maintained. Finally, the fibers were allowed to cool naturally to ambient temperature.

#### 2.2.2. Rapid Thermal Deposition Technique

The ZIF-8 membrane was synthesized by first preparing two solutions: 1.32 g of Zn(OAc)_2_ and 1 g of 2 mIm, each dissolved in 15 mL of a 2:1 DMAc/DI-water mixture. Solution 1 was gradually added to Solution 2 under continuous stirring for 15 min, forming a turbid mixture. The α-alumina fibers were dip-coated in the mixture at a linear speed of 5 mm/min with a 10 s immersion time, using a syringe pump for controlled application. The coated fibers were then heat-treated at 473 K for 15 min, followed by slow cooling. The ZIF-8 membrane underwent a 24 h solvent exchange with MeOH and was air-dried at room temperature. Prior to gas permeation testing, the membrane was degassed at 393 K for 8 h to ensure solvent removal and structural stability.

#### 2.2.3. Graphene Oxide (GO) Synthesis

Graphite oxidation was carried out using a modified Hummer’s method [16]. In this process, 1 g of graphite was combined with 150 mL of concentrated sulfuric acid (98%) and 3.5 g of KMnO_4_, with the reaction maintained at 35 °C for 1 to 20 h. Afterward, distilled water and hydrogen peroxide were gradually introduced while cooling the reaction in an ice bath to control its progress. The reaction vessel was sealed with aluminum foil to minimize moisture exposure. The resulting mixture was filtered, and the solid product was thoroughly washed with 10% hydrochloric acid. The final product was freeze-dried to eliminate any remaining moisture.

#### 2.2.4. Deposition of GO on α-Alumina Hollow Fiber

An aqueous GO solution was prepared by dispersing GO powder in deionized water. The GO powder was exfoliated using sonication for a specific duration to achieve the desired dispersion. The concentration of the GO solution was varied between 0.5 mg/mL and 20 mg/mL to control the properties of the final film. The α-alumina hollow fiber was briefly immersed in the GO solution, followed by drying at ambient temperature. The thickness of the resulting GO film was adjusted by altering the concentration of the GO solution, with higher concentrations yielding thicker films. The PVDF fiber, now coated with the GO film, was then subjected to further drying at 70 °C for 2 h to ensure complete removal of residual moisture.

### 2.3. Characterization

X-ray diffraction (XRD) patterns were recorded using a D2 Phaser diffractometer, Bruker, Massachusetts, USA at room temperature, employing Cu Kα radiation (λ = 0.154 nm) over a scanning range of 5–40° 2θ. Surface and cross-sectional SEM images were captured using a GeminiSEM 300, ZEISS, Jena, Germany, with all membrane samples pre-treated by Pt sputter coating (Q150R Plus-Rotary Pumped Coater, Quorum Technologies, Lewes, UK. Elemental analysis via EDX was performed using a XFlash 6I30, Bruker, Billerica, MA, USA system attached to the SEM. Additionally, a Park XE-100 AFM, Park systems, Korea system was used to examine the three-dimensional structure and generate numerical profiles.

### 2.4. Permeation Test

The permeance of the C_3_H_6_/C_3_H_8_ gas mixture was measured using gas chromatography. An equimolar C_3_H_6_/C_3_H_8_ mixture with a flow rate of 20 SCCM was used as the feed, and 20 SCCM of argon flowed on the permeate side as the sweep gas. The permeance *F_i_* of the membrane is calculated as Equation (1):

(1) $$F_{i}=\frac{R_{i}}{A{\times ∆P}_{i}}$$

where *R_i_* is the mole rate of component *i* (mol/s), *A* is the area of the membrane (m^2^), and Δ*P_i_* is the partial pressure passing through the membrane.

Selectivity (*α_i,j_*) for component *i,j* is calculated as Equation (2):

(2) $$\alpha_{i,j}=\frac{y_{i}/y_{j}}{x_{i}/x_{j}}$$

where *x_i_*and *x_j_* are the mole fractions of *a* and *b* in the feed stream, and *y_i_* and *y_j_* are the mole fractions in the permeate stream.

### 2.5. Zinc Oxide Atomic Layer Deposition

All processes were performed at 120 °C for 5 to 50 cycles. After placing the sample in the reactor, the chamber was evacuated to approximately 10⁻^2^ torr. Prior to depositing ZnO, nitrogen gas flowed at 50 SCCM for 12 s to remove any remaining precursor inside the reactor. A 0.3 s pulse of diethylzinc was then introduced, followed by purging with 50 SCCM of nitrogen for 15 s. After the purge, a 0.3 s pulse of H_2_O was applied, followed by a 15 s nitrogen purge at 50 SCCM. This process was repeated 5 to 50 times. Upon completing the deposition, a final nitrogen flow of 500 SCCM for 180 s was applied to ensure the complete removal of any remaining precursors from the reactor.

## 3. Result and Discussion

**Figure 1:**
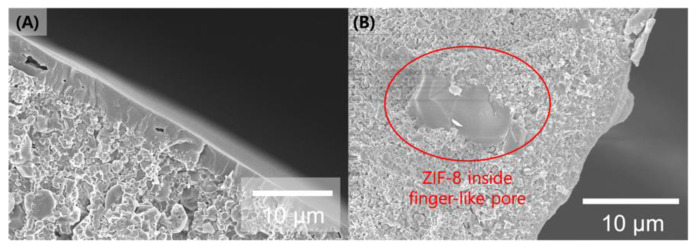
Cross-sectional SEM images of (**A**) ZIF-8 membranes on bare α-alumina hollow fiber supports using RTD, and (**B**) deep penetration of ZIF-8 into the finger-like pores of the α-alumina support.

As mentioned, we reported a simple synthesis process for fabricating α-alumina hollow fiber-supported ZIF-8 membranes using Rapid Thermal Deposition (RTD), which minimizes precursor consumption and reduces processing time [13]. This method relies on the evaporation of the ZIF-8 precursor solution absorbed into the pores of an alumina substrate to form a selective layer. As shown in Figure 1, the ZIF-8 membrane was well-formed; however, directly immersing the bare α-alumina substrate into the ZIF-8 precursor solution can lead to deeper penetration of the precursors, resulting in thick (>2 μm) membranes (Figure 1A) or undesired deep penetration of the ZIF-8 layer inside the finger-like pores of the α-alumina support (Figure 1B). This structure negatively affects the gas separation performance of the membrane. To address this issue, we employed a graphene oxide (GO) gutter layer as a barrier for the hierarchical fabrication of polycrystalline ZIF-8 membranes in this study.

**Figure 2:**
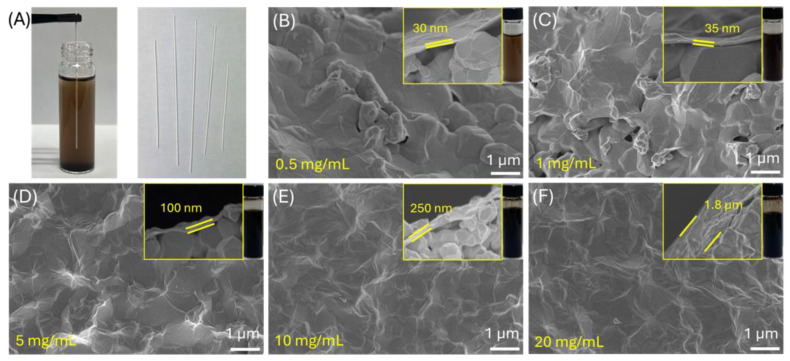
(**A**) Photograph of GO solution and bare α-alumina hollow fibers before coating. (**B**–**F**) Cross-sectional SEM images of GO-coated α-alumina fibers with varying GO concentrations: (**B**) 0.5 mg/mL, (**C**) 1 mg/mL, (**D**) 5 mg/mL, (**E**) 10 mg/mL, and (**F**) 20 mg/mL.

Images of the graphene oxide (GO) solution and bare alumina hollow fibers are shown in Figure 2A, illustrating how GO flakes naturally adhere to the surface of an α-alumina hollow fiber. The fiber was completely dried at 120 °C. Afterward, the fibers were briefly immersed in an aqueous GO solution, where the GO flakes adhered to the fiber’s outer surface due to capillary forces within the α-alumina pores. The oxygen-functional groups on both GO and α-alumina likely contributed to this adhesion. Figure 2B–F depict GO-layered α-alumina supports with thicknesses ranging from 30 nm to 1.8 μm, depending on the concentration of GO used. A 100 nm thick GO layer was chosen for the GO gutter layer formation, as thicker layers could reduce gas permeability, while thinner ones might not provide full surface coverage. Atomic force microscopy (AFM) in Figure S1 confirms that the GO gutter-layered alumina support exhibited a surface roughness below 130 nm, with the GO layer uniformly covering the α-alumina surface, leaving no exposed alumina areas.

**Figure 3:**
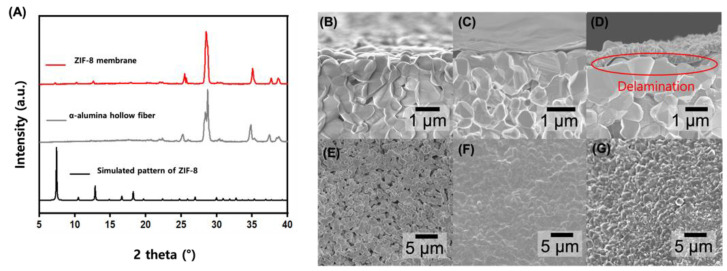
(**A**) pXRD patterns of the ZIF-8 membrane on GO-layered α-alumina support, bare α-alumina hollow fiber, and simulated ZIF-8 pattern. Cross-sectional SEM images showing (**B**) bare α-alumina support, (**C**) GO-coated α-alumina support, and (**D**) as-synthesized GO@ZIF-8 layer, with the delaminated interface between the GO layer and α-alumina. (**E**–**G**) Corresponding top-view SEM images of (**E**) bare α-alumina, (**F**) GO-coated support, and (**G**) GO@ZIF-8 layer.

The GO-treated α-alumina hollow fiber support was then used for RTD membrane growth. Briefly, the hollow fiber was placed vertically in a vial attached to a syringe pump. The RTD solution, containing Zn^2+^ and 2 mIm in a 2:1 DMAc/H₂O mixture, was injected into the vial at a rate of 5 mm/min. After a brief dwell time, the solution was withdrawn at the same rate, allowing some of the solution to be absorbed through the hollow fiber by osmotic pressure. The fiber was then heated to 200 °C, causing solvent evaporation and rapid ZIF-8 crystallization. The presence of a ZIF-8 layer was confirmed by the similarity between the pXRD results and the simulated ZIF-8 pattern, while the weak peak intensity is attributed to the thinness of the layer (Figure 3A). Figure 3B–D present cross-sectional SEM images of the bare alumina support (Figure 3B), the alumina support after GO layer deposition (Figure 3C), and the as-synthesized GO@ZIF-8 layer (Figure 3D). The corresponding top-view SEM images are shown in Figure 3E–G. A uniform 300 nm thick ZIF-8 layer was grown on the outer surface of the GO-layered α-alumina support. However, a delaminated interface between the GO layer and the support was observed, resulting in a defective membrane. Consequently, the membrane exhibited no gas separation performance. This is likely because, although the GO layer contains sufficient functional groups such as hydroxyl, epoxy, carboxyl, and carbonyl groups, the α-alumina support lacks these functional groups due to the high sintering temperature (>1000 °C). Therefore, it can be concluded that a simple GO flake coating could not provide a strong interaction with the α-alumina support.

To overcome the delamination issue, zinc oxide (ZnO) ALD was performed on the GO-layered α-alumina support. The ALD process for ZnO involves alternating exposure to two chemical precursors: diethylzinc (DEZ) as the zinc source and water as the oxygen source. During ALD, DEZ reacts with the surface hydroxyl groups present in both the GO layer and the α-alumina support [17]. The thickness of the ZnO layer increases proportionally as the number of ALD cycles increases. After performing ZnO ALD cycles ranging from 5 to 50 cycles, the surface characteristics were examined (Figure S2). Up to 20 ALD cycles, a smooth surface was observed with no visible differences among the cycles; however, once the ALD cycles reached 50 (Figure S2D), ZnO particles were detected on the surface of the GO layer.

**Figure 4:**
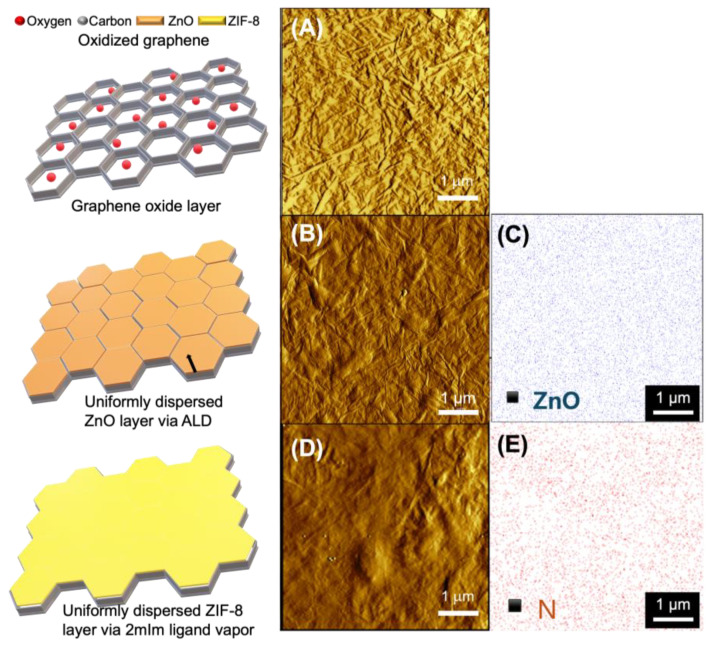
(**A**) GO layer surface before ALD treatment, showing surface wrinkles. (**B**) GO surface after 20-cycle ZnO ALD treatment. (**C**) EDX mapping of Zn showing uniform distribution on the GO surface after 20 ALD cycles. (**D**) GO surface after post-treatment with 2 mIm vapor. (**E**) EDX mapping of the nitrogen atom. The illustrations in each column were designed to facilitate understanding.

To investigate changes in the surface properties of GO, the GO layer and the 20-cycle ALD-coated GO layer were analyzed using AFM amplitude mode, and the results are shown in Figure 4A,B. The surface wrinkles caused by GO were reduced, and no agglomerated ZnO particles were observed. This suggests that ZnO was well dispersed in the form of a layer. Additionally, Energy Dispersive X-Ray (EDX) analysis confirmed that Zn was uniformly distributed on the surface (Figure 4C). Therefore, the 20-cycle ZnO ALD-treated GO-layered α-alumina was chosen and then exposed to the vapors generated by the sublimation of 2 mIm at 120 °C. It has been previously reported that ZnO can be converted into ZIF-8 upon exposure to the 2 mIm precursor at elevated temperatures.

After post-treatment with the 2 mIm vapor organic precursor, the GO surface became smoother (Figure 4D), and uniformly distributed nitrogen atoms were detected (Figure 4E), indicating a well-dispersed ZIF-8 layer. Considering the low ZIF density and assuming full conversion to ZIF-8, the levels of Zn detected within the GO layer after 2 mIm vapor treatment are sufficient to create strong interactions both between GO layers and between the GO layer and the α-alumina support.

**Figure 5:**
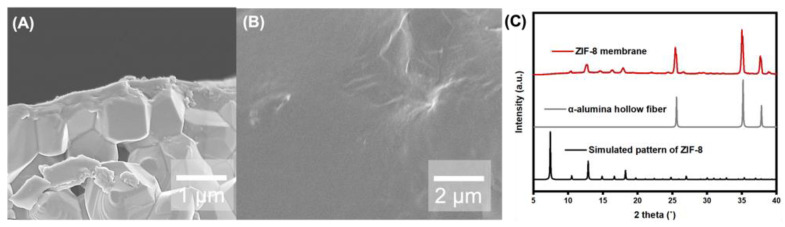
(**A**) Cross-sectional and (**B**) top-view SEM image of ZIF-8 membrane grown on 2 mIm vapor-treated GO-layered α-alumina support, and (**C**) corresponding pXRD patterns.

Finally, RTD membrane growth was conducted on the 2 mIm vapor-treated GO-layered α-alumina support. Compared to the membrane synthesized on the bare support, which had a thickness of approximately 2 μm (Figure 1A), the membrane thickness on the GO-layered support post-treated with 2 mIm vapor was significantly reduced to about 300 nm (Figure 5A), resulting in an approximately 6-fold decrease in thickness. The top-view surface SEM image and PXRD pattern confirmed the presence of a uniformly grown, defect-free ZIF-8 layer (Figure 5B,C). It is noted that, compared with the simulated spectra, the PXRD peak intensity is slightly reduced. This reduction may not be entirely attributed to the decrease in the ZIF-8 layer thickness; it could also result from partial carbonization or collapse of the ZIF-8 framework during the RTD process [18]. Interestingly, in the GO layer without ZnO ALD pre-treatment, crystalline particles and grain boundaries were observed, whereas the membrane with ZnO ALD pre-treatment exhibited a smooth, featureless surface. Notably, the delamination between the GO layer and the alumina support, which was previously observed in the RTD membrane synthesis on the bare GO-layered support, was no longer present. This change is likely due to the conversion of ZnO, originally present between the GO layer and the alumina surface, into ZIF-8, which enhanced the interfacial bonding between the two layers. Even after sonicating the 2 mIm vapor-treated GO-layered α-alumina support for over 5 min, no delamination between the GO layer and the alumina was observed. Additionally, no deep penetration of the ZIF-8 layer was observed. Finally, it should be noted that we performed an additional experiment in which ZnO ALD was directly applied to the alumina support without the GO layer, followed by RTD; however, no significant improvement compared to the untreated RTD membrane was achieved.

**Figure 6:**
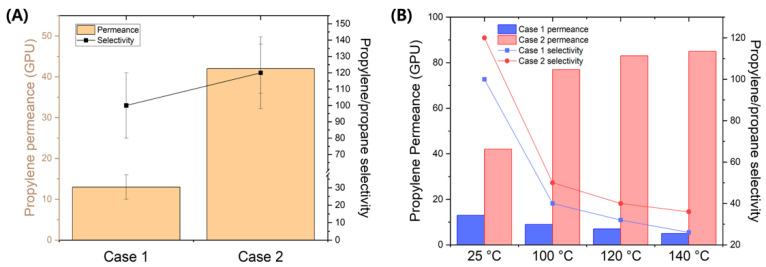
(**A**) Propane/propylene separation performance of ZIF-8 membranes on non-coated α-alumina (Case 1) and GO-coated α-alumina (Case 2). (**B**) Temperature dependence of propylene permeance and propylene/propane selectivity for the non-coated α-alumina (Case 1) and GO-coated α-alumina (Case 2).

To clarify the microstructural advantages of the GO-coated α-alumina support, propane/propylene separation performance was evaluated for ZIF-8 membranes on both non-coated α-alumina (Case 1) and GO-coated α-alumina (Case 2). The separation efficiency of an equimolar propane/propylene mixture was tested using the steady-state Wicke–Kallenbach method at 293 K, with results averaged from three independent samples for each case (Figure 6A). Reflecting their improved structure, the Case 2 membranes demonstrated significantly higher propylene permeance (~42 GPU), about three times greater than Case 1, due to the thinner membrane. Furthermore, despite the typical trade-off between permeance and selectivity, Case 2 membranes achieved higher selectivity (~120), suggesting minimal inter-crystalline defects.

Interestingly, the GO-layered alumina-supported ZIF-8 membrane demonstrated an increase in propylene permeance with rising temperature (Figure 6B). As the temperature increased from 25 °C to 100 °C, the permeability of propylene nearly doubled, though at higher temperatures, the rate of increase became more gradual. This increase in permeability was also observed for propane gas, resulting in a decrease in propylene/propane selectivity from 120 to 38. Typically, the adsorption of propylene in ZIF-8 is an exothermic process, where adsorption effects tend to dominate over diffusion in the solution-diffusion mechanism [19]. Consequently, as shown in Figure 6B for the Case 1 membrane, a decrease in propylene permeance with increasing temperature would generally be expected. However, the observed increase in permeance suggests that the GO layer may be altering the membrane’s microstructure, potentially enhancing diffusion rates at higher temperatures. There are reports indicating that the presence of graphene oxide (GO) in ZIF-8 membranes can significantly influence gas permeance, particularly in response to temperature variations [20,21]. The incorporation of GO is known to improve the permeability of ZIF-8 membranes by modifying their microstructure and porosity. As temperature rises, the permeance of gases through ZIF-8/GO composite membranes often increases, likely due to enhanced molecular diffusion and potential changes in the flexibility or pore dynamics of the framework at elevated temperatures. A comparative table of the membrane performance with previous studies has been provided in Table S2. While the membrane did not outperform cutting-edge counterparts, the observed increase in permeance with rising temperatures and the successful fabrication of a thin film via the GO layer suggest that this approach holds promise for future research. With further optimization, there is potential for significant performance enhancement, making this concept valuable for ongoing developments in the field.

## 4. Conclusions

In this study, we successfully developed ZIF-8 membranes supported on GO-coated α-alumina hollow fibers using a combination of Rapid Thermal Deposition (RTD) and ZnO Atomic Layer Deposition (ALD) techniques. By incorporating a GO gutter layer, we were able to mitigate the issue of excessive precursor penetration and control membrane thickness, significantly reducing it to approximately 300 nm. This approach also eliminated delamination issues between the GO layer and the α-alumina support, resulting in a more robust and defect-free membrane. The GO-coated ZIF-8 membranes exhibited a three-fold increase in propylene permeance compared to membranes fabricated without the GO layer. Furthermore, the enhanced selectivity (~120) indicates that the GO layer effectively minimized inter-crystalline defects. The membranes demonstrated temperature-dependent performance, with propylene permeance nearly doubling as the temperature increased from 25 °C to 100 °C, while selectivity declined as propane permeance increased at higher temperatures. This suggests that the GO layer may influence the membrane’s microstructure, enhancing diffusion rates and gas transport under varying thermal conditions. Overall, the combination of GO coating and ZnO ALD pre-treatment presents a promising strategy for fabricating high-performance ZIF-8 membranes with improved gas separation properties, particularly for challenging separations like propane/propylene. This approach offers potential for scalable and efficient membrane-based separations, providing a pathway towards more energy-efficient and sustainable industrial processes.

## Data Availability

The original contributions presented in the study are included in the article/supplementary material, further inquiries can be directed to the corresponding author.

## Supplementary Materials

The following supporting information can be downloaded at: https://www.mdpi.com/article/10.3390/membranes14100216/s1, Figure S1: (A) AFM height mode image of GO-layered alumina support, and (B) corresponding line scan height information; Figure S2: SEM images of GO surface with varying ZnO ALD cycles: (A) bare GO, (B) 5 cycles, (C) 20 cycles, and (D) 50 cycles (inset shows ZnO particles); Table S1: Dope composition and conditions and for dry-jet-wet spinning of α-alumina hollow fibers; Table S2: Summary of membrane performance for propylene/propane separation in the literature. References [22,23,24,25,26,27,28,29,30,31,32,33,34,35,36,37] are cited in Supplementary Materials.
