# Supplementary material for: Controlled Growth of ZIF-8 Membranes on GO-Coated α-Alumina Supports via ZnO Atomic Layer Deposition for Improved Gas Separation
Source: Membranes (Basel). 2024 Oct 14;14(10):216. doi: 10.3390/membranes14100216 (PMC11509257; doi:10.3390/membranes14100216)
Supplement: Supplementary file 1 [file membranes-14-00216-s001.zip › membranes-3236725-supplementary.pdf]

membranes

Supporting Materials

Controlled Growth of ZIF-8 Membranes on GO-Coated α-Alumina Supports via ZnO Atomic Layer Deposition for Improved Gas Separation

Nahyun Lee, Yun-ho Ahn, Jaheon Kim and Kiwon Eum *

School of Chemical Engineering, Soongsil University, Seoul 06978, Republic of Korea
* Correspondence: kiwon.eum@ssu.ac.kr

Table S1. Dope composition and conditions and for dry-jet-wet spinning of α-alumina hollow fibers.

**Dope composition (PES/NMP/Al_2O_3/PVP) (wt %)**	7/38/54/1
**Dope flow rate (ml/h)**	120
**Bore fluid**	DI-water
**Bore fluid˚ flow rate (ml/h)**	80
**Air gap (cm)**	3
**Operating temperature (K)**	298
**Quench bath temperature (K)**	298

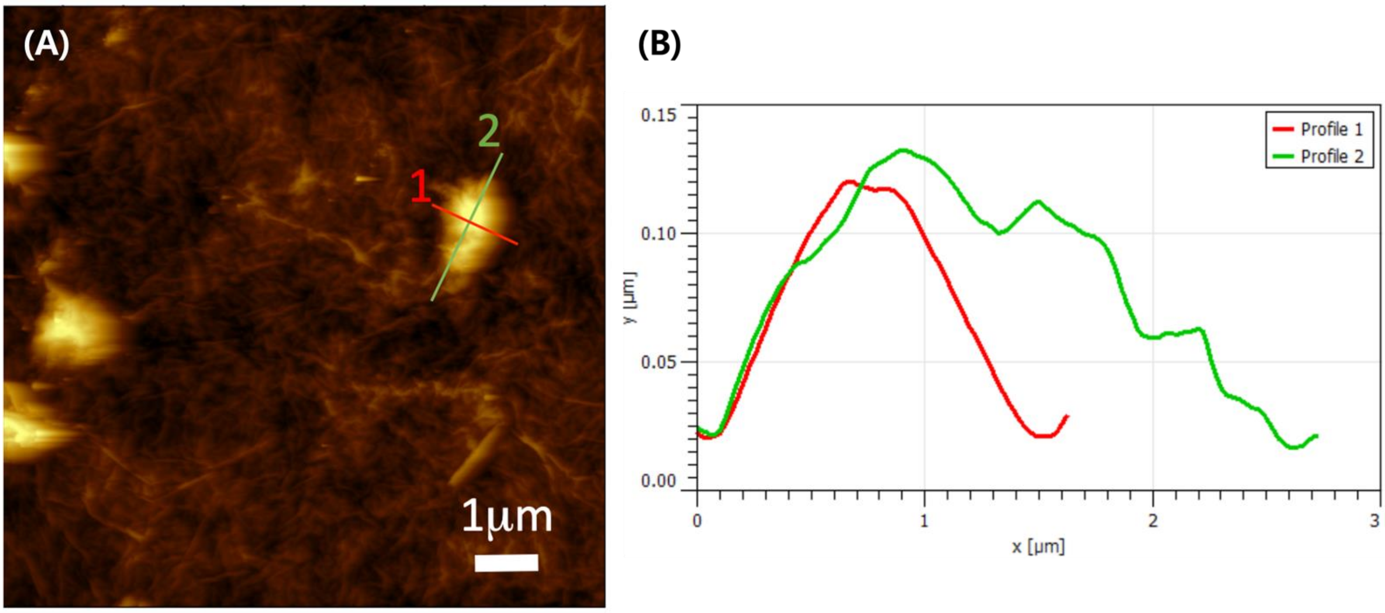

Figure S1. (**A**) AFM height mode image of GO-layered alumina support, and (**B**) corresponding line scan height information.

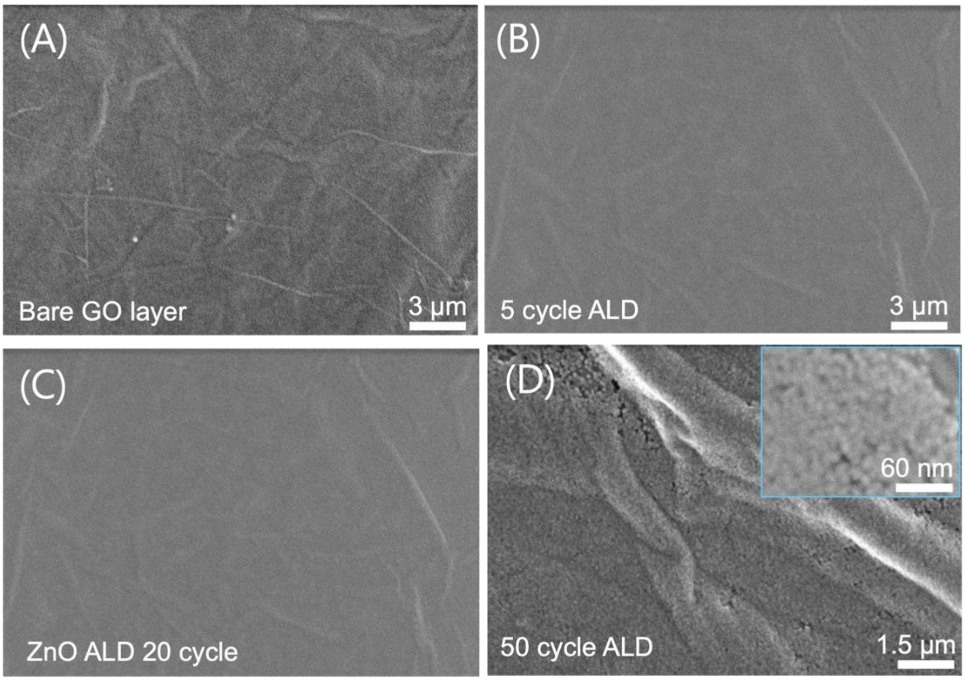

Figure S2. SEM images of GO surface with varying ZnO ALD cycles: (**A**) bare GO, (**B**) 5 cycles, (**C**) 20 cycles, and (**D**) 50 cycles (inset shows ZnO particles).

Table S2. Summary of membrane performance for propylene/propane separation in the literature.

Membrane Type	Support Type	Temperature(°C)	C_3H_6 Permeance (GPU)	C_3H_6/C_3H_8 Selectivity	Reference
GO-coated ZIF-8	Hollow fiber	25	90	120	This work
GO-coated ZIF-8	Hollow fiber	140	18	38	This work
ZIF-8	a-Al_2O_3 disk	120	32.0	30.1	[22]
ZIF-8		120	45.1	26.3	
ZIF-8	Hollow fiber	25	6.87	57	[23]
ZIF-8	Hollow fiber	25	116	6.9	[24]
ZIF-8		25	15.5	7.2	
ZIF-8		25	35.8	20.0	
ZIF-8	a-Al_2O_3 disk	25	42.9	30.8	[25]
ZIF-8	Hollow fiber	25	31	10	[26]
ZIF-8		25	38	27	
ZIF-8		120	12	21	
ZIF-67	a-Al_2O_3 disk	25	460	85	[27]
ZIF-8/ZIF-67		25	370	209	
PPO	film	32	0.1	4.3	[28]
PIM-1	film	35	57.2	2.8	[29]
PIM-CD-1 wt%			86.3	2.5	
PIM-CD-2 wt%			100.6	2.4	
6FDA-mPD	film	35	0.01	10	[30]
6FDA-TrMPD	film	50	1.5	11	[31]
6FDA-DDBT			0.04	27	
6FDA-ODA			0.02	11	
PDMS/Silica-5%	film	0	47.8	4.0	[32]
PDMS/Silica-10%			38.5	4.5	
PDMS/Silica-15%			31.7	5.2	
PDMS/Silica-20%			15	6.8	
PDMS/Silica-25%			9.7	7.3	
PDMS/Silica-30%			4.3	10.2	
Carbon derived PI-LPSQ10	Hollow fiber	35	3.4	22	[33]
Carbon derived PI	film		4.8	10	
Carbon derived from PAEK/Azide (50:50)-450ºC	film	35	0.03	31	[34]
Carbon derived from PAEK/Azide (50:50)-550ºC			0.02	48	
Carbon derived from BPDADDBT/DABA asymmetric (400°C)	Hollow fiber	100	1.8	3	[35]
Carbon derived from BPDADDBT/DABA asymmetric (600°C)			51	12	
Ag-X	a-Al_2O_3 disk	30	231	94.2	[36]
NaX			245	5.55	
FAU	a-Al_2O_3 disk	25	203	7.9	[37]
